# Second-generation *p*-values: Improved rigor, reproducibility, & transparency in statistical analyses

**DOI:** 10.1371/journal.pone.0188299

**Published:** 2018-03-22

**Authors:** Jeffrey D. Blume, Lucy D’Agostino McGowan, William D. Dupont, Robert A. Greevy

**Affiliations:** 1 Associate Professor, Department of Biostatistics, Vanderbilt University School of Medicine, Nashville, Tennessee, United States of America; 2 PhD Candidate, Department of Biostatistics, Vanderbilt University School of Medicine, Nashville, Tennessee, United States of America; 3 Professor, Department of Biostatistics, Vanderbilt University School of Medicine, Nashville, Tennessee, United States of America; University of Illinois-Chicago, UNITED STATES

## Abstract

Verifying that a statistically significant result is scientifically meaningful is not only good scientific practice, it is a natural way to control the Type I error rate. Here we introduce a novel extension of the *p*-value—a second-generation *p*-value (*p*_*δ*_)–that formally accounts for scientific relevance and leverages this natural Type I Error control. The approach relies on a pre-specified interval null hypothesis that represents the collection of effect sizes that are scientifically uninteresting or are practically null. The second-generation *p*-value is the proportion of data-supported hypotheses that are also null hypotheses. As such, second-generation *p*-values indicate when the data are compatible with null hypotheses (*p*_*δ*_ = 1), or with alternative hypotheses (*p*_*δ*_ = 0), or when the data are inconclusive (0 < *p*_*δ*_ < 1). Moreover, second-generation *p*-values provide a proper scientific adjustment for multiple comparisons and reduce false discovery rates. This is an advance for environments rich in data, where traditional *p*-value adjustments are needlessly punitive. Second-generation *p*-values promote transparency, rigor and reproducibility of scientific results by *a priori* specifying which candidate hypotheses are practically meaningful and by providing a more reliable statistical summary of when the data are compatible with alternative or null hypotheses.

## Introduction

*P*-values abound in the scientific literature. They have become the researcher’s essential tool for summarizing when the data are incompatible with the null hypothesis. Although *p*-values are widely recognized as imperfect tools for this task, the impact of their flaws on scientific inference remains hotly debated [1–5]. The debate over the proper use and interpretation of *p*-values has stymied and divided the statistical community [6–14]. Recurring themes include the difference between statistical and scientific significance, the routine misinterpretation of non-significant *p*-values, the unrealistic nature of a point null hypothesis, and the challenges with multiple comparisons. With no widely-accepted alternative to promote, statisticians are left to tweak the manner in which *p*-values are applied and interpreted [11,12]. Some have even suggested that the problem lies with instruction: *p*-values are fine, they are just widely misused [15,16]. After a century of widespread adoption in science, with their flaws and advantages well-known, it is time for an upgrade.

The purpose of this paper is to introduce a novel and intuitive extension that better serves the *p*-value’s intended purpose. We call this upgrade a second-generation *p*-value. Second-generation *p*-values are easy to compute and interpret. They offer improved inferential capability, e.g. it is now possible for the data to indicate support for the null hypothesis. They control the Type I error naturally, forcing it to zero as the sample size grows. This, in turn, offsets Type I Error inflation that results from multiple comparisons or multiple examinations of accumulating data. Findings identified by second-generation *p*-values are less likely to be false discoveries than findings identified by classical *p*-values. Consequently, second-generation *p*-values do not require ad-hoc adjustments to provide strict error control and this improves power in studies with massive multiple comparisons. They also implicitly codify good research practice: the smallest effect size of scientific relevance must now be specified before looking at results. This prevents the inevitable rationalization that accompanies the post-hoc interpretation of mediocre results that have been deemed statistically significant. This singular change alone will improve rigor and reproducibility across science.

Our examples (Section 3) were selected from a wide range of contexts to highlight the broad utility of this new tool. We will not dwell on the well-known drawbacks of classical *p*-values [11,12,13,14]. The frequency properties of second-generation *p*-values are the same or better than traditional *p*-values. These technical details, along with supplementary exposition, can be found in the supplementary materials (S1 and S2 Files). A distinguishing feature of second-generation *p*-values is that they are intended as summary statistics that indicate when a study has met its a priori defined endpoint: the observed data support only alternative hypotheses or only null hypotheses.

Given the complexity surrounding the interpretation and computation of *p*-values, and the plethora of ad-hoc statistical adjustments for them, the reader is forgiven for any pre-emptive statistical fatigue, pessimism, or skepticism. After all, every statistical adjustment for multiple comparisons boils down to nothing more than ranking the *p*-values and picking a cutoff to determine significance. While each method offers its own preferred cut-off, the core value judgement—the ranking—remains the same. Second generation *p*-values, however, change that ranking; they favor results that are both scientifically relevant and statistically significant. For example, Section 3 presents an application where a Bonferroni correction yields 264 genes of interest from a study of 7128 candidate genes where 2028 had an unadjusted *p*-value of 0.05 or less. An application of the second-generation *p*-value also yields 264 gene findings (their second-generation *p*-value is 0), ensuring the same Type I Error control. However, 82 (31%) of those genes fail to meet the Bonferroni criteria. The difference is both fascinating and striking, and is due to the second-generation *p*-value’s preference for scientific relevance (which in this case amounts to a preference for clinically relevant fold changes in expression levels).

### 1.1 Illustration of approach

**Fig 1:**
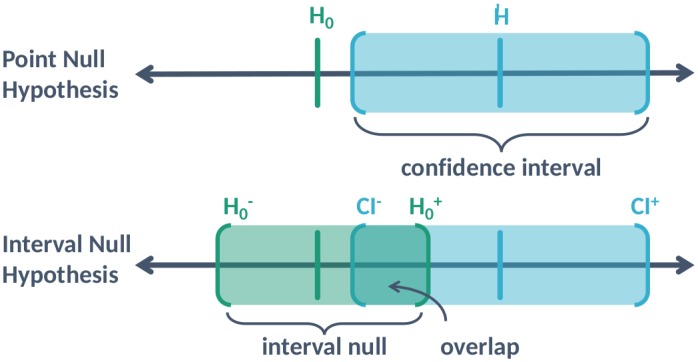
Illustration of a point null hypothesis, *H*_0_; the estimated effect that is the best supported hypothesis, $\hat{H}$; the 95% confidence interval (CI) for the estimated effect [*CI*^−^, *CI*^+^]; and the interval null hypothesis $\left[H_{0}^{-},\;H_{0}^{+}\right]$.

The top diagram of Fig 1 depicts an estimated effect, typically the best supported hypothesis $\hat{H}$, its 95% confidence interval (CI), and the traditional point null hypothesis, *H*_0_. The CI contains all the effect sizes that are supported by the data at the 95% level; we will refer to it as the set of data-supported hypotheses. If the null hypothesis is well outside of the interval, the *p*-value is very small or near zero. If the CI just barely excludes the null hypothesis, the *p*-value will be slightly less than 0.05. When the CI contains the null hypothesis, the *p*-value will be larger than 0.05. The *p*-value grows to 1 as the null hypothesis approaches the center of the CI.

Now imagine that the null hypothesis is a contiguous set—an interval—rather than just a single point, as depicted in the bottom diagram of Fig 1. The interval null is the set of effects that are indistinguishable from the null hypothesis, due to limited precision or practicality. For example, the null hypothesis “no age difference” might be re-framed as “no age difference of more than 365 days”, the latter being what we really mean when we say two people are the same age (e.g., they are both 45). An interval null always exists, even if it is narrow.

When a 95% CI is entirely contained within the null interval, the data support only null hypotheses (this is the traditional benchmark for showing statistical equivalence). When the CI and null set do not overlap, the data are said to be incompatible with the null. Lastly, when the null set and confidence interval partially intersect, the data are inconclusive. Thus, the degree of overlap conveys how compatible the data are with the null premise. The second-generation *p*-value is the fraction of overlap multiplied by a small-sample correction factor. We define it formally in Section 2. *In a very real sense*, *the second-generation p-value is nothing more than the codification of today’s standards for good scientific and statistical practice*.

### 1.2 Interval vs. point null hypothesis

The formal acknowledgement of an interval null hypothesis has important consequences for how statistical methods are applied and understood in science. Any point hypothesis, while mathematically convenient, represents a statistical hypothesis so precise it can never be confirmed with finite data. For null hypotheses, this high level of specificity can complicate inference. For example, when a point null hypothesis is “rejected”, it can be the case that there are other point hypotheses, practically indistinguishable from the point null, that remain well supported by the data. The solution is to use an interval null hypothesis. These are constructed by incorporating information about the scientific context—such as inherent limits on measurement precision, clinical significance, or scientific significance—into statistical hypotheses that are stated *a priori*. The tag ‘scientifically relevant’ or ‘clinically significant’ is reserved for effects (hypotheses) that are non-trivial and meaningful, i.e., beyond the interval null hypothesis. Measurement precision can always be used to establish a lower bound on scientifically relevant hypotheses, as it makes little sense to ponder effect sizes that are smaller than the detectable limit. The interval null should contain, in addition to the precise point null hypothesis, all other point hypotheses that are practically null and would maintain the scientific null premise. While the point null may be numerically distinct, all the hypotheses in the interval null are considered scientifically equivalent to the null premise.

## Definition and computation

### 2.1 Formula

Let *I* represent the interval of hypotheses for a scalar parameter that are best supported by the data—an unadjusted 95% CI for example—and let *H*_0_ represent the interval null hypothesis. If *I* = [*a*, *b*] where *a* < *b* are real numbers, then its length is |*I*| = *b* − *a*. The second-generation *p*-value, denoted by *p*_*δ*_, is defined as

$$p_{\delta }=\begin{array}{c} \frac{\left|I\cap H_{0}\right|}{\left|I\right|}\\ \; \end{array}\;\;\;\times \;\;\text{max}\left\{\frac{\left|I\right|}{2\left|H_{0}\right|},1\right\}$$ (1)

where *I* ∩ *H*_0_ is the intersection, or overlap, between intervals *I* and *H*_0_. The subscript *δ* signals the reliance of the second-generation *p*-value on the interval null. Numerically, *δ* represents the half-width of the interval null hypothesis. The value of *δ* is driven by scientific context and should be specified prior to conducting the experiment. The first term in Eq (1) is the fraction of best supported hypotheses that are also null hypotheses. The second term is a small-sample correction factor, which forces the second-generation *p*-value to indicate inconclusiveness when the observed precision is insufficient to permit valid scientific inferences about the null hypotheses.

As described here, *p*_*δ*_ is the length of the intersection between the two intervals, divided by the length of the interval estimate, multiplied by the correction factor. When the interval estimate is sufficiently precise, defined here as when |*I*| < 2|*H*_0_|, the second-generation *p*-value is just the overlap fraction, |*I* ∩ *H*_0_|/|*I*|. When the interval estimate is very wide, |*I*| > 2|*H*_0_|, the second-generation *p*-value reduces to 0.5 × |*I* ∩ *H*_0_|/|*H*_0_|, which is bounded by ½.

Definition (1) readily extends to multiple dimensions to accommodate parameter vectors. In that case, |*I*| would represent an area or volume. Neither interval is required to be symmetric or of finite length. Although the vast majority of intervals in the literature are ‘two-sided’ with finite length, pathologies are possible when neither interval has finite length. If intervals *I* and *H*_0_ overlap but neither has finite length, e.g., overlapping one-sided intervals, the second-generation *p*-value will be zero or one, depending respectively on whether |*I* ∩ *H*_0_| is finite or infinite (Remark 1 in S1 File). Note that any interval estimate, from a likelihood support interval to a Bayesian credible interval, could be used in place of the 95% confidence interval (more on this point in Section 2.6). Inferential denomination, computational ease, and desired frequency properties could inform this choice.

### 2.2 A simple example

**Fig 2:**
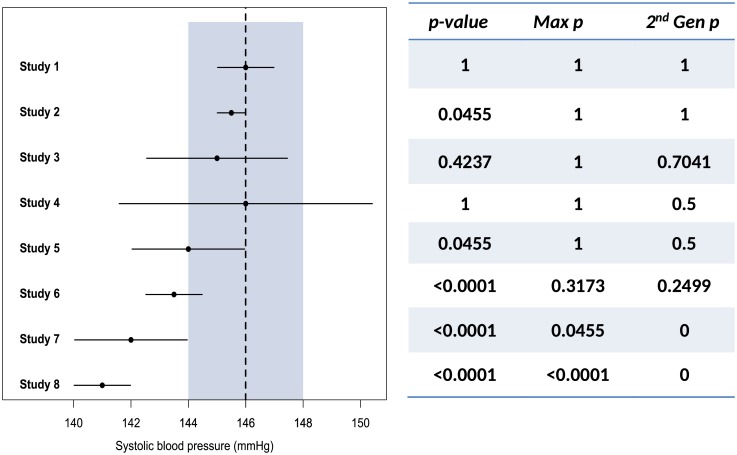
Forest plot and p-value statistics of mock results from 8 studies of systolic blood pressure. Here the point null is 146 mmHg, indicated by the vertical dashed line, with an indifference zone, or interval null hypothesis, from 144 mmHg to 148 mmHg shaded in blue-grey.

When measuring systolic blood pressure (SBP), the currently accepted recommendation is to report SBP to the nearest 2 mmHg when using analog or mercury devices [17,18]. Blood pressure dials indicate even numbers and changes less than 2 mmHg are not clinically actionable. Fig 2 and Table 1 display results from 8 mock studies.

**Table 1:** Mock results from 8 studies of systolic blood pressure.

Study	Mean (SE)	95% CI		2^nd^ Gen. *p*-value (*p*_*δ*_)	Maximum *p*-value	Traditional *p*-value
		Lower	Upper			
1	146 (0.5)	145.02	146.98	1	1	1
2	145.5 (0.25)	145.01	145.99	1	1	0.0455
3	145 (1.25)	142.55	147.45	0.7041	1	0.4237
4	146 (2.25)	141.59	150.41	0.5	1	1
5	144 (1)	142.04	145.96	0.5	1	0.0455
6	143.5 (0.5)	142.52	144.48	0.2449	0.3173	<0.0001
7	142 (1)	140.04	143.96	0	0.0455	<0.0001
8	141 (0.5)	140.02	141.98	0	<0.0001	<0.0001

Table 1 reports the mean SBP, the 95% CI, the second-generation *p*-value based on *δ* = 2 mmHg, the maximum *p*-value over all possible null hypotheses in the range *H*_0_: 144 ≤ *μ* ≤ 148, and the traditional *p*-value for testing *H*_0_: *μ* = 146.

For example, in Study 3, the upper confidence bound is 142.55 and the lower confidence bound is 147.45. The interval null, *μ*_0_ ± *δ* = [144, 148], has length |*H*_0_| = 2*δ* = 4 mmHg. So *p*_*δ*_ is

$$p_{\delta }=\frac{\left(147.45-144\right)}{\left(147.45-142.55\right)}\left(1\right)=0.7041$$ (2)

A *p*_*δ*_ of 0.7 means the data are inconclusive with a slight or weak favoring of null hypotheses. Intuitively, it means that 70% of the data-supported hypotheses are null hypotheses. Study 4 is an example where the correction factor comes into play. The length of the confidence interval, 8.82, is more than twice the length of the null interval, 4. Therefore, *p*_*δ*_ is:

$$p_{\delta }=\frac{\left(148-144\right)}{\left(150.41-141.59\right)}\frac{\left(150.41-141.59\right)}{2\left(148-144\right)}=0.5$$ (3)

A *p*_*δ*_ of 0.5 means the data are strictly inconclusive. The role of the correction factor is discussed in Section 2.5.

The second-generation *p*-values best describe Fig 2. Study 1 is an example where the data only support null effects. Study 2 demonstrates the paradox of statistical and scientific significance. Both *p*_*δ*_, and the maximum *p*-value seem to account for this paradox. However, *p*_*δ*_ has more desirable attributes in other circumstances, such as in Studies 3 to 6. Study 4 has a traditional *p*-value and maximum *p*-value of 1, but the data are clearly inconclusive. The second-generation *p*-value reflects this, with a value of 0.5, allowing for a more nuanced interpretation of ‘inconclusive’. In studies where the point estimate falls in the null range, but the confidence interval extends beyond (e.g., studies 3, 4, and 5) the maximum *p*-value does not properly convey what the data are saying, and the traditional *p*-value does not account for the range of null values, or the precision of the measurement tool. Studies 7 and 8 illustrate the case when the confidence interval is fully beyond the null space.

### 2.3 Interpretation

Consider a general interval null hypothesis, say *H*_0_: *μ*_0*a*_ ≤ *μ* ≤ *μ*_0*b*_. The second-generation *p*-value, *p*_*δ*_, has the following properties:

1. *p*_*δ*_ is a proportion; a number between zero and one, inclusive.
2. *p*_*δ*_ is the fraction of data-supported hypotheses that are null hypotheses and therefore compatible with the null premise.
3. When *p*_*δ*_ = 0, the data only support hypotheses that are scientifically or clinically meaningful, i.e., those that are meaningful alternative hypotheses.
4. When *p*_*δ*_ = 1, the data only support null hypotheses, i.e., those that are *not* scientifically or clinically meaningful.
5. When *p*_*δ*_ = ≈ 1/2, the data are strictly inconclusive. The degree of inconclusiveness is represented by *p*_*δ*_ itself. For example, *p*_*δ*_ = 1/8 and *p*_*δ*_ = 7/8 both represent the same degree of inconclusiveness, but the balance between alternative and null hypotheses is reversed.
6. *p*_*δ*_ has improved error rate control when the interval estimate *I* is a 100(1 − *α*) % CI. Under any null hypothesis within the interval null, the probability of observing *p*_*δ*_ = 0 is less than or equal to *α*. This probability converges to zero as the sample size grows. Under any hypotheses beyond the interval null, the probability of observing *p*_*δ*_ = 0 converges to one as the sample size grows.

The interpretation of *p*_*δ*_ may appear similar to that of a posterior probability of the null hypothesis. However, *p*_*δ*_ is strictly not a posterior probability and it is not an estimate of the probability of the null hypothesis. Rather, *p*_*δ*_ is simply the observed fraction of data-supported hypotheses that are null hypotheses; it is a descriptive statistic—a simple proportion. Essential elements of a proper posterior computation, namely knowledge that some hypotheses are better supported than others and the degree to which some hypotheses inside the interval null should be favored over others, are not needed. Specification of the latter is controversial because it does not depend on the data at hand. Second-generation *p*-values are descriptive; they indicate when the study has generated data that rule out null or alternative hypotheses.

The third property does not hold for traditional *p*-values; this is why *statistical significance is not scientific significance*. The fourth property is strictly false for classical *p*-values; a *p*-value larger than the Type I Error probability *α* is considered inconclusive. Large *p*-values never “support” the null hypothesis; they just indicate the lack of strong evidence against it. This is the *absence of evidence is not evidence of absence* conundrum. The fifth property is also not strictly true for traditional *p*-values; when non-significant, the *p*-value is to be interpreted as inconclusive despite the temptation to do otherwise. A welcome feature of second-generation *p*-values is that they distinguish between data that are inconclusive (0 < *p*_*δ*_ < 1) and data that are compatible with null hypotheses (*p*_*δ*_ = 1). This ability is sorely needed in practice.

The sixth property is a major improvement. Unlike its predecessor, the second-generation *p*-value converges to zero or one as the sample size grows, which means the procedure is inferentially consistent in the limit (Remark 2 in S1 File). Why this happens is interesting and intuitive (Remark 3 in S1 File). The take-home message is that their frequency properties are no worse than those of the interval estimates upon which the second-generation *p*-value is based, and are often improved in moderate to large samples. A detailed examination of the frequency properties of second-generation *p*-values is included in the supplement (Remarks 10–18 in S1 File).

### 2.4 The delta gap

It can be helpful to have a way of ranking two studies that both have second-generation *p*-values of zero (*p*_*δ*_ = 0). One way to do this is to use the *delta-gap*, which is the distance between the intervals in *δ* units. Recall that *δ* is the half-width of the interval null hypothesis. If the CI was shifted to the right of the null interval, the delta-gap would be $(CI^{-}-H_{0}^{+})/\delta$. Scaling by *δ* makes it unit free. The delta-gap ranking favors extremes in effect size, which is a logical complement to the second-generation *p*-value. Remember that second-generation *p*-values provide a quick and easy marker of when a study reaches a natural endpoint, i.e., when the data are compatible with only null hypotheses (*p*_*δ*_ = 1) or only alternative hypotheses (*p*_*δ*_ = 0). Two studies with equal second-generation *p*-values do not necessarily represent equal amounts of statistical evidence. For example, their likelihood functions may not be proportional. The same is true of classical *p*-values, of course [11,12].

### 2.5 Role of small-sample correction factor

The small-sample correction factor comes into play when the intervals overlap and the range of data supported hypotheses is more than twice that of the indifference zone, i.e., when |*I*| > 2|*H*_0_|. Because the width of the interval estimate *I* will shrink as the sample size grows, the correction factor comes into play more often in small samples.

The second-generation *p*-value is based on the proportion of data-supported hypotheses that are null, or practically null, hypotheses. This is written as |*I* ∩ *H*_0_|/|*I*|. When the data are very imprecise, this proportion alone can be misleading. To see this, consider the case when |*I*| ≫ |*H*_0_| and the two intervals completely overlap. The proportion alone would be small, indicating that the data favor alternative hypotheses even though every possible null hypothesis is just as well supported. Clearly the data are inconclusive, and the correction factor resets the proportion to ½ to indicate this.

The correction factor allows second-generation *p*-values greater than ½ to reliably represent degrees of compatibility with the null hypotheses. When the correction factor is applicable, the second-generation *p*-value is bounded above by ½, which is achievable only when the null interval is entirely contained within the interval estimate. When the intervals fail to intersect, the relative degree of precision is immaterial and *p*_*δ*_ = 0 because there are no hypotheses in common.

How often will the correction factor come into play? It depends on a number of factors. For planned experiments, a general benchmark is that the correction factor plays a role when the power to detect the smallest meaningful hypothesis drops below 16% (Remark 4 in S1 File). That is, it comes into play only for studies that are severely underpowered to detect meaningful effect sizes.

### 2.6 Choosing an interval estimate

In this paper, we choose 95% CIs for the interval estimate *I*. However, the definition of a second-generation *p*-value is not so exclusive. Any interval estimate, from a likelihood support interval to a Bayesian credible interval [19, 20], could be used. Different interval estimates will impart different frequency properties to the second-generation *p*-value. The interpretation and usage of *p*_*δ*_ would remain unchanged as long as *I* represented, in some sense, a set of best supported hypotheses. Our preference is to use a 1/8 likelihood support interval (SI). SIs have a well-established evidential interpretation, are not dependent on the sample space, and are otherwise well aligned with the *p*-value’s *raison d’être* [21,22]. A 1/8 likelihood support interval—the benchmark for moderate strength of evidence—is equivalent to an unadjusted 95.9% CI in many regular cases, so their distinction is largely interpretative [11,21]. Because confidence intervals have near universal familiarity, and because support intervals are essentially unadjusted confidence intervals, we decided not to feature support intervals in this exposition. This choice should not be mistaken for an implicit endorsement of confidence intervals (Remark 5 in S1 File).

## Applications

The first example contrasts the behavior of second-generation *p*-values with classical methods when examining differential gene expression in 7128 candidate genes. The second is a comparison of Kaplan-Meier survival curves, often done in biomarker development, where the multiple comparisons issue is hidden by design. Additional examples, for 2x2 contingency tables (Remark 8 in S1 File) and regression models (Remark 9 in S1 File), can be found in the supplement.

### 3.1 Multiple comparisons: Leukemia Microarray study

The ALL-AML Leukemia Microarray Study [23] involved 72 leukemia participants. Forty-seven had acute lymphoblastic leukemia (ALL) and 25 had acute myeloid leukemia (AML). The study sought to identify genes with differential expression between the two types of leukemia. A microarray analysis was conducted on 7128 candidate genes and two-group t-tests were performed on the cleaned and standardized log_10_ expression levels of each gene. Here the effect scale is the difference in the logarithm of gene expression levels or fold-change (log-ratio). Typically, a fold change must be greater than 2 to draw interest, implying our interval null should be from ½ to 2, or -0.3 to 0.3 the log_10_ scale. This demarcation does not imply that fold-change is a surrogate for scientific importance. It simply means that an estimated fold-change must meet some minimum criterion to be considered interesting. It is the small effects that, when statistically significant, tend to be false discoveries (See Section 4.2).

**Fig 3:**
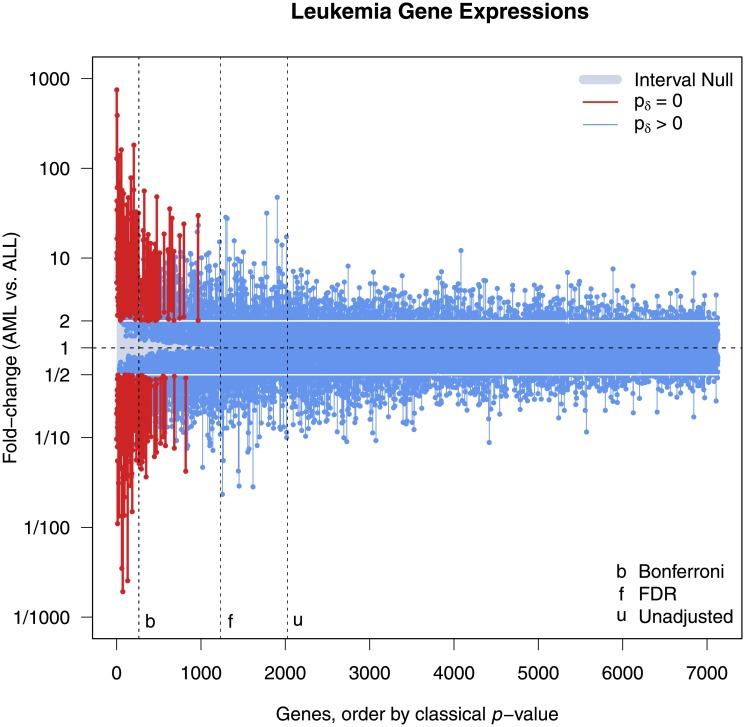
Display of 95% confidence intervals for gene specific fold-changes (AML vs. ALL) in the gene expression levels of patients from the Leukemia Microarray study [23]. All 7128 genes are sorted on the x-axis by classical *p*-value rank. Interval null hypothesis (blue-grey zone) shows all absolute fold changes between ½ and 2. Red genes have a second-generation *p*-value of 0, blue genes do not. Vertical dashed lines show various traditional *p*-value cutoffs at the 0.05 level (Bonferroni, Benjamini-Hochberg false discovery rate, and unadjusted).

Fig 3 displays 7128 unadjusted 95% CIs for fold change when the genes are ordered by their classical *p*-value rank. The vertical dotted lines show the Bonferroni cutoff, the empirical false discovery rate (FDR or q-value) cutoff [24], and the unadjusted *p*-value cutoff that results from *α* = 0.05. Only 264 genes remain ‘statistically significant’ after a Bonferroni correction; 1233 have an estimated FDR less than *α*; and 2028 genes have an unadjusted classical *p*-value less than *α*. The indifference / null zone is shaded in a blue-grey color. The 229 CIs in red have a second-generation *p*-value of zero (*p*_*δ*_ = 0); none of the fold changes in these CIs are between ½ and 2. Note that these findings cannot be ordered on the basis of the second-generation *p*-value alone. CIs that extend into the blue-grey zone have a *p*_*δ*_ > 0; each of them indicates that the data support fold changes between ½ and 2. Under a global null hypothesis, the Bonferroni and the second-generation *p*-value approaches have nearly the same apparent Type I Error rate for findings: 0.037 (= 264/7128) vs. 0.032 (= 229/7128).

**Fig 4:**
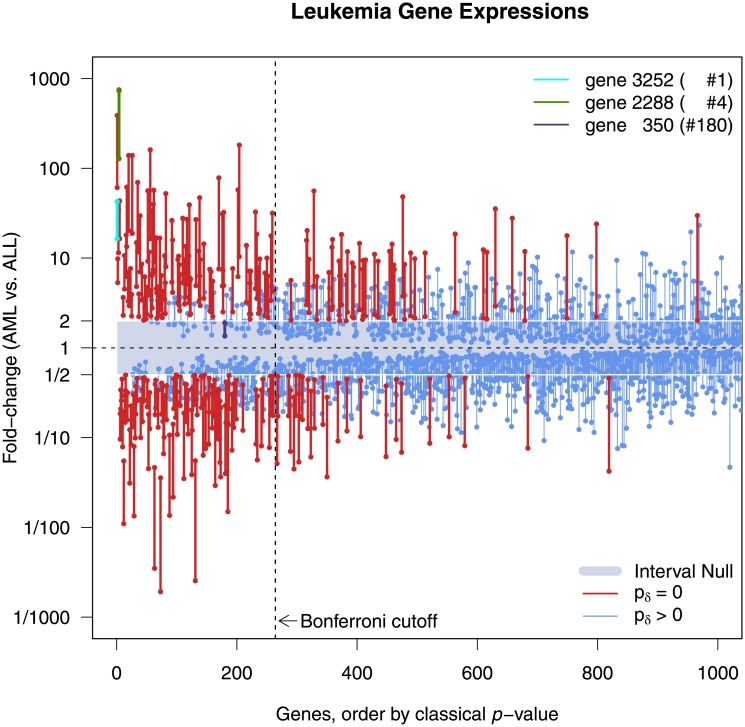
Top 1,000 ranked genes from the Leukemia Microarray study [23]. Display of 95% confidence intervals for gene specific fold-changes (AML vs. ALL). Genes sorted by classical *p*-value rank. Interval null hypothesis (blue-grey zone) shows all absolute fold changes between ½ and 2. Red genes have a second-generation *p*-value of 0, blue genes do not. Genes 3252 (light blue) and 2288 (green) have a second-generation *p*-value of 0, while gene 350 (dark blue) has a second-generation *p*-value of 1.

Fig 4 displays the top 1,000 genes according to their *p*-value rank. There are 229 genes with *p*_*δ*_ = 0 (i.e., red confidence intervals). Of these, 65 are missed by Bonferroni. The indifference zone need only be narrowed slightly, 1/1.915 to 1.915, to also have exactly 264 second-generation *p*-values that are 0. Yet even then, Bonferroni still misses 82 of them. Table 2 provides a cross-tabulation for this comparison. Consider gene #6345, whose *p*-value of 0.0033 is ranked 966^th^ but has a 95% CI for fold change of 2.02 to 29.74, and gene #350, whose *p*-value of 9.02×10^−7^ is ranked 180^th^ but has 95% CI for fold change of 1.36 to 1.94. Bonferroni finds the second gene, where the data support only trivial fold changes, and misses the first, where the data support only meaningful fold changes. The second-generation *p*-value does just the opposite.

**Table 2:** Cross-tabulation of second generation p-values with Bonferroni corrected p-values.

	1/2 < Fold Change < 2 (*δ* = 0.3)		1/1.915 < Fold Change < 1.915 (*δ* = 0.282)	
	***p***_***δ***_ = **0**	***p***_***δ***_ > **0**	***p***_***δ***_ = **0**	***p***_***δ***_ > **0**
***p***_***bon***_ **< 0.05**	164	100	182	82
***p***_***bon***_ **> 0.05**	65	6799	82	6782
**Total**	229	6899	264	6864

The empirical FDR criterion could be lowered to 2.45% (slightly more than half) and still capture the same 229 genes with *p*_*δ*_ = 0. However, it would also capture an additional 737 genes for which *p*_*δ*_ > 0. All either overlap the null interval substantially or are contained in the null interval. As we will see in section 4.2, the actual FDR depends on a number of factors, and it does not necessarily make sense to apply the same FDR criterion to all genes. Moreover, the false discovery rate for the second-generation *p*-value is less than that for the classical *p*-value (or comparable to, depending on the significance level). All this means is that adjusting the *p*-value cutoff is not likely to help much, because it leaves the original rank ordering unchanged.

Second-generation *p*-values are selecting a fundamentally different set of candidate genes. An important point is that this set cannot be identified by standard methods where selection is based only on *p*-value ranking. To illustrate this, the supplement details what happens in the leukemia example as the indifference zone shrinks to zero (Remark 6 in S1 File). Consider also, the delta-gap ranking among the 229 genes where *p*_*δ*_ = 0. Gene #2288, for example, has the 4^th^ lowest traditional *p*-value, but has the largest a delta gap at 6 (computed on log_10_ scale). Gene #3252 has the lowest traditional *p*-value, but has a delta gap of 3 (10^th^ largest). Remark 18 in S1 File details these computations.

### 3.2 Survival

**Fig 5:**
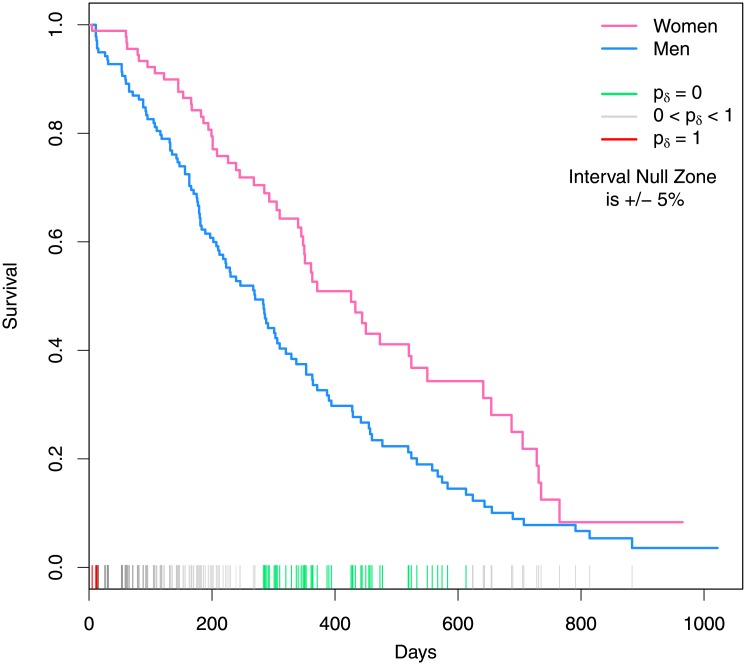
Survival in patients with advanced lung cancer from the North Central Cancer Treatment Group study. Kaplan-Meier survival curves by gender (blue for men, pink for women). Rug plot on x-axis displays second-generation *p*-values for the difference in survival time. Green ticks indicate incompatibility with null hypotheses; red indicate compatibility; gray indicate inconclusive results.

Survival data on patients with advanced lung cancer are available from the North Central Cancer Treatment Group [25]. Fig 5 displays Kaplan-Meier survival curves for women (pink) and men (blue). A log-rank test (p = 0.0013) indicates the curves are statistically different *somewhere*. Second-generation *p*-values tells us where. Suppose survival differences greater than 5% are of interest, implying an interval null from -0.05 to 0.05 percentage points.

The rug plot on Fig 5 shows color coded second-generation *p*-values at every observed difference in the Kaplan-Meier curves. The ticks are green when *p*_*δ*_ = 0 (real difference), red when *p*_*δ*_ = 1 (no meaningful difference, if any), and shades of grey when inconclusive. It is easy to see that the curves differ by at least 5 percentage points between 300 and 600 days. Otherwise the apparent differences are inconclusive except for at the beginning when the curves are essentially the same. Remark 7 in the supplement details these calculations (Remark 7 in S1 File). There are 139 points where the curves can be compared, but this is immaterial to the second-generation *p*-value. The log-rank test avoids the implicit multiple comparisons issue, but at the cost of being non-specific. It cannot identify where the curves are different, nor is the test itself unique [26].

A common alternative approach is to use a Cox model with gender as a covariate and rely on the proportional hazards assumption. This yields an estimated hazard ratio of 1.7 (men to women) with a 95% CI of 1.23 to 2.36. Assuming that that hazard ratios between 0.9 and 1.1 are not clinically interesting, we have a *p*_*δ*_ = 0. Hence the data, incompatible with null hypotheses, suggest a meaningful difference in lung cancer risk between men and women.

### 3.3 Additional examples and applications

The supplement contains an application of second-generation *p*-values for assessing an odds ratio in a 2x2 contingency table (Remark 8 in S1 File) and for assessing whether the data are compatible with the removal of a set of predictors from a linear regression model (Remark 9 in S1 File).

## Frequency properties of second generation p-values

Second-generation *p*-values generally maintain the kind of error rate control that science has become accustomed to. Moreover, they are a more reliable inferential tool than classical *p*-values. A technical treatment of this topic is provided in supplemental remarks 10 through 18 (Remarks 10–18 in S1 File). Here we briefly recount the key findings.

### 4.1 Behavior of second-generation *p*-values under presumed conditions

An examination of the stochastic behavior of second-generation *p*-values is revealing. Upon collecting data, a second-generation *p*-value may indicate compatibility with the alternative hypothesis (*p*_*δ*_ = 0), compatibility with null hypotheses (*p*_*δ*_ = 1), or inconclusiveness (0 < *p*_*δ*_ < 1). How often these events occur, under various null and alternative hypotheses, is of interest when designing a study.

When a null hypothesis is true, we do not observe *p*_*δ*_ = 0 often. In fact, *P*(*p*_*δ*_ = 0|*H*_0_) ≤ *α* and often *P*(*p*_*δ*_ = 0|*H*_0_) ≪ *α*. Here (1 − α) denotes the coverage probability of the interval estimate. Unlike the Type I Error rate of hypothesis testing, *P*(*p*_*δ*_ = 0|*H*_0_) shrinks to zero as the sample size grows so long as *H*_0_ is in the interior of the null interval. This is partly why second-generation *p*-values are advantageous in multiple comparison settings. At the edges, the probability remains constant at *α*.

When a true alternative hypothesis is outside the null interval, we do not often observe *p*_*δ*_ = 1. This event cannot occur until the interval estimate is narrow enough to be contained by the interval null, i.e. when the sample size is large. Here too, *P*(*p*_*δ*_ = 1|*H*_1_) ≤ *α* for any *H*_1_ outside the null interval with *P*(*p*_*δ*_ = 1|*H*_1_) ≪ *α* for *H*_1_ not at the edge of the null interval. The *P*(*p*_*δ*_ = 1|*H*_1_) also shrinks to zero as the sample size grows.

The least desired outcome is an inconclusive second-generation *p*-values, 0 < *p*_*δ*_ < 1. A common occurrence in small studies, the probability of this happening is written as *P*(0 < *p*_*δ*_ < 1|*H*). This probability reaches its maximum, 1 − *α*, when the true hypothesis *H* is on the edge of the null interval. As the true hypothesis *H* moves away from the edge, the probably decreases to zero. For example, the supplement (S1 File) shows that *P*(0 < *p*_*δ*_ < 1|*H*) ≈ 0.15 when *H* is *three standard errors* from the edge. Hence, sample size is a means of controlling this probability. In this sense, the probability of observing an inconclusive *p*_*δ*_ is analogous to the Type II Error rate of hypothesis testing.

Second-generation *p*-values do sacrifice some power to characterize findings into three categories. The criteria for rejecting the null is more stringent, and inconclusive results must be separated from those supporting the null. However, the reduction in power is typically less than that caused by popular multiple comparison adjustments such as Bonferroni. As such, in contexts with massive multiple comparisons and varying standard errors, the second-generation *p*-value outperforms Bonferroni because its error rate profile is generally superior (Remark 21 in S1 File).

Although this improved inferential clarity comes with a real cost, it also yields a critical advantage: the second-generation *p*-value has a lower false discovery rate than a comparable hypothesis test. That is, second-generation *p*-values are more reliable tools than classical *p*-values.

### 4.2 Reliability of an observed second-generation *p*-value

Once data are collected and the second-generation *p*-value is computed, the long-run behavior of second-generation *p*-values becomes irrelevant. The relevant quantity is the probability that the observed results, say *p*_*δ*_ = 0 or 1, are mistaken. This tendency, which we will refer to as the reliability of second-generation *p*-values, is captured by the false discovery rate (FDR) *P*(*H*_0_|*p*_*δ*_ = 0) and the false confirmation rate (FCR) *P*(*H*_1_|*p*_*δ*_ = 1).

A straightforward application of Bayes rule allows us to compute these rates as

$$P\left(H_{0}|p_{\delta }=0\right)={\left[1+\frac{P(p_{\delta }=0|H_{1})}{P(p_{\delta }=0|H_{0})}r\right]}^{-1}\;\text{and}\;P\left(H_{1}|p_{\delta }=1\right)={\left[1+\frac{P\left(p_{\delta }=1|H_{0}\right)}{P\left(p_{\delta }=1|H_{1}\right)}\frac{1}{r}\right]}^{-1}$$ (4)

where *r* = *P*(*H*_1_)/*P*(*H*_0_). The dependence of these rates on the design probabilities *P*(*p*_*δ*_ = 0|*H*_1_), *P*(*p*_*δ*_ = 0|*H*_0_), *P*(*p*_*δ*_ = 1|*H*_0_), *P*(*p*_*δ*_ = 1|*H*_1_), and prior probability ratio *r*, is instructive. Eq (4) explains how a study design influences the reliability of subsequent inference (Remark 19 in S1 File).

Every second-generation *p*-value inherits its reliability from the study design and the original odds of the alternative hypothesis. This reliability varies with the alternative hypothesis in question. As the distance between the alternative and null grows, the FDR and FCR decrease. However, the wide range of possible alternatives makes it hard to summarize the FDR and FCR with a single number. Summarization is even more of a problem in high-dimensions, such as our leukemia example, where every finding would ideally be accompanied by its estimated reliability.

**Fig 6:**
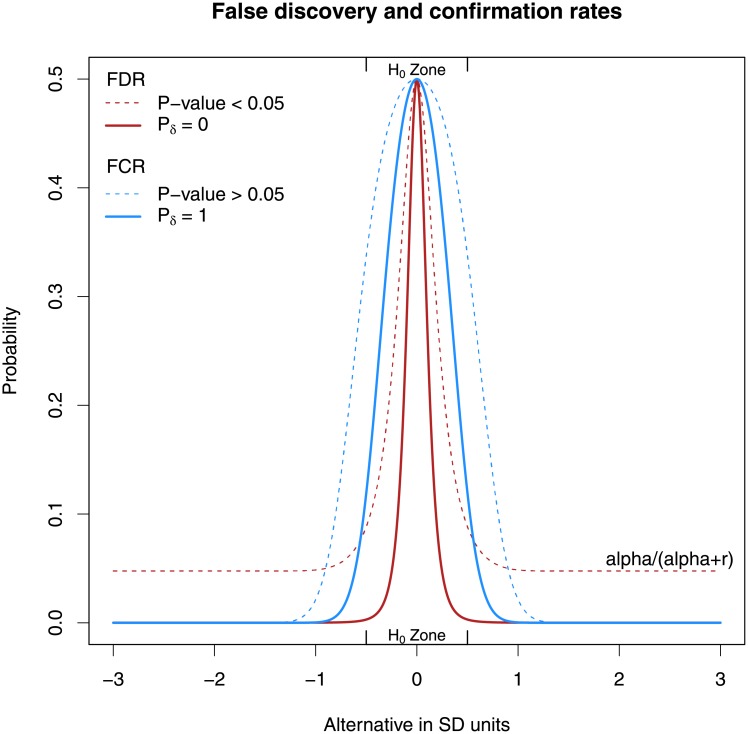
Illustration of the false discovery rate (red) and false confirmation rate (blue) for second-generation *p*-values (solid lines). The false discovery rate (red) and false non-discovery rate (blue) from a comparable hypothesis test are shown as dotted lines. This example uses r = 1, α = 0.05, δ = σ/2, and n = 16, but the ordering of the curves is quite general.

Fig 6 displays the FDR and FCR (solid lines) when the width of the interval null is equal to one standard deviation (*δ* = *σ*/2) and the sample size is large enough to permit *p*_*δ*_ = 1. Included for comparison are the false discovery rate and false non-discovery rate of a comparable hypothesis test (dotted lines). These rates are *P*(*H*_0_│*Rejected H*_0_) = [1 + *r*(1 − *β*)/*α*]^−1^ and *P*(*H*_1_│*Failed to Reject H*_0_) = [1 + (1 − *α*)/*βr*]^−1^. The FDR and FCR for second-generation *p*-values are generally smaller than their hypothesis testing counterparts. Remark 20 in S1 File displays this relationship for various sample sizes.

In principle, the reliability of any inferential summary ought to be reported along with the summary itself. Neither is truly sufficient on its own. However, reporting the reliability can be difficult to do in practice for the reasons noted above. And while standards for reporting the FDR and FCR deserve further investigation and discussion, the key message is that second-generation *p*-values decrease the FDR and FCR from classical *p*-values.

We note that while estimates further from the point null are more reliable in the sense that their false discovery rates are lower, this does not mean that they are more ‘important’, more ‘relevant’ or more ‘meaningful’ in a scientific sense. A low false discovery rate means only that the results are more likely to replicate under identical conditions. The cause for this replication could be a true scientific finding or an unknown experimental flaw. Only careful investigation into the validity of the scientific experiment can determine which it is.

## Comments

More than a century of experience tells us that *p*-value usage, despite its flaws, will persist. Science desires an easily digestible and reliable summary of whether the data are compatible with null or alternative hypotheses. *Second-generation p-values are tailor made for this role*. They are easy to apply and interpret; they can be used in conjunction with frequentist, Bayesian or Likelihood methods; and they exhibit excellent frequency properties. Moreover, they eliminate the haggling over ad-hoc adjustments to *p*-values that have become a real challenge in high-dimensional and data-rich settings.

A well-known problem with classical *p*-values is that they can be small and yet also be associated with confidence intervals that include hypotheses or parameter values that are essentially null. In genomic studies with many thousands of variants, this has led to the detection of hundreds of variants with spurious significance. By introducing an interval null hypothesis of scientifically equivalent null hypotheses, second-generation *p*-values avoid the vast majority of the spuriously significant findings. By better reflecting the true nature of the null hypothesis in mathematical terms, statistical inference works better for science. Also, the width of the interval null need not be large, as the benefits of second-generation *p*-values will eventually be realized as the sample size grows.

Some challenges remain. The statistical properties of this new tool need to be explored and detailed in a variety of settings. Our preliminary findings detailed here, and in the web-supplement (S1 File), indicate that second-generation *p*-values tend to have excellent behavior overall. Nonetheless, this should be explored in detail (See our “Frequently Asked Questions” in S2 File). In practice, disagreement on the width of the interval null (*δ*) will require re-calculation of the second-generation *p*-value. To facilitate this, we encourage the reporting and discussion of the interval estimates upon which the second-generation *p*-value is based. We remind the reader that the second-generation *p*-value is not intended to be the final product, but rather a quickly digested summary measure. Understanding the force and implication of statistical results will always require attention to the details of the statistical analysis.

Regardless of the analysis, caution should always be used when interpreting statistical findings. Just because a result is statistically less likely to be a ‘false discovery’ does not mean that the result is more ‘important’, ‘relevant’ or ‘meaningful’ in a scientific sense. It means simply that the results are more reliable and more likely to replicate under identical circumstances, even if those circumstances are in some way flawed. A statistical hypothesis is a specific, precise mathematical statement about an unknown parameter. And statistical hypotheses assume a probability model to perform computations. If that model fails, then the associated statistical hypotheses often fail to be valid translations of the scientific hypotheses. This is, in fact, one of the most common criticisms of point null hypothesis testing; the point null is almost never *exactly* correct (employing an interval null hypothesis addresses this concern directly). In this paper, we assumed that the broader statistical model holds because this is a routine assumption for *p*-value based inference. However, the robustness of the second-generation *p*-value to model misspecification is an important topic that deserves attention.

We anticipate that the major challenge in implementing second-generation *p*-values will be encouraging researchers to define a scientifically relevant finding *prior to examining the data*. While this is already routine in some areas of biomedicine (e.g. clinical trials), it is not even on the radar in others. Nevertheless, second-generation *p*-values are a clear improvement over classical *p*-values. They are between 0 and 1. They have a straightforward interpretation. A second-generation *p*-value of 0 is properly interpreted as favoring alternative hypotheses. A second-generation *p*-value of 1 is properly interpreted as favoring null hypotheses. A second-generation *p*-value between 0 and 1 favors both types of hypotheses and is properly interpreted as inconclusive. The error rates for second-generation *p*-values are bounded by *α* and converge to 0. Adjustments for multiple comparisons are obviated, and lower false discovery rates accompany observed results. In short, *the second-generation p-value achieves the inferential properties that many scientists hope*, *or believe*, *are attributes of the classic p-value*. Using second-generation *p*-values can only improve rigor, reproducibility and transparency across science.

## Supporting information

S1 File Supplementary remarks (with figures). (PDF)

S2 File Frequently asked questions. (PDF)
