# Supplementary material for: Second-generation p-values: Improved rigor, reproducibility, & transparency in statistical analyses
Source: PLoS One. 2018 Mar 22;13(3):e0188299. doi: 10.1371/journal.pone.0188299 (PMC5863943; doi:10.1371/journal.pone.0188299)

Supplementary Materials Part 1 (S1): Remarks (with figures)

Title: Second-generation *p*-values: improved rigor, reproducibility & transparency in statistical analyses

To appear in PLOS ONE

Authors: Jeffrey D. Blume*, Lucy D'Agostino McGowan, William D. Dupont, Robert A. Greevy Jr.
*Correspondence to: j.blume@vanderbilt.edu

Contents:
Supplementary Remarks Pages: S1 – S12
Supplementary References Pages: S13
Supplementary Figures Pages: S14 – S19

Remark 1. Naturally, neither I nor H_0 may contain the entire parameter space. Other pathologies are easily rectified. For example, if intervals I and H_0 overlap, but $I \subseteq H_0$, i.e., I is a subset of H_0, then $p_\delta = 1$ regardless of the length of the intervals. The problem arises when the intersection is finite, $|I \cap H_0| < \infty$, but both intervals are not. For example we might have $I = [c, \infty)$ and $H_0 = (-\infty, d]$ with $c < d$ real numbers. Now we have $|I \cap H_0| = d - c$ and we could argue that $|I|/|H_0| = 1$. But $|I \cap H_0|/|I|$ is arguably zero, whereas $p_\delta = 0$ seems inappropriate here because the intervals have a finite set of hypothesis in common. A practical and realistic solution is to simply truncate I at effects that are not possible to observe in practice.

Remark 2. Note that the procedure is inferentially consistent for all null and alternative hypothesis that are not on the boundary of the indifference zone. When the true hypothesis is exactly on the boundary of the interval null, say at $\mu_0 + \delta$, the second-generation *p*-value will have essentially the same frequency properties as a classical hypothesis test. As a result, the Type I Error rate of α will remain constant as a function of the sample size and the procedure is no longer inferentially consistent in the limit. That is, it will be wrong $100\alpha\%$ of the time regardless of the sample size.

Remark 3. An underappreciated fact of statistical inference is that over 99% of Type I Errors occur between 1.96 to 4 *standard errors* from the null. Because $2 * (\Phi[4] - \Phi[1.96])/0.05 = 0.9986$ where $\Phi[x] = P(Z \leq x)$ is the standard normal cumulative distribution function. In moderate to large samples, alternative hypotheses in this region are very close to the null hypothesis in absolute units and seldom represent a practically different model than the null. Because p_δ

excludes alternative hypothesis that are scientifically indistinguishable from the point null hypothesis – even if statistically significant – the rate of Type I Errors is dramatically reduced.

Remark 4. The exact relationship will depend on circumstances, but this simple case provides a good guide. Let E be the margin of error (half width) from a $(1-\alpha)100\%$ CI with sample size n. Also, let n be the sample size from a two-sided hypothesis test with size α and power $1-\beta$ to detect an alternative that is δ units from the null hypothesis. Assuming the variance is constant, we have that

$$n = \left(\frac{Z_{1-\alpha/2} + Z_{1-\beta}}{\delta}\right)^2 = \left(\frac{Z_{1-\alpha/2}}{E}\right)^2 \quad \text{(S1)}$$

If we let δ represent the smallest change of scientific interest, then $2\delta = |H_0|$ and $|I| = 2E$ and we have that

$$|I| = \left(\frac{Z_{1-\alpha/2}}{Z_{1-\alpha/2} + Z_{1-\beta}}\right)|H_0| \quad \text{(S2)}$$

where $Z_{1-\alpha/2} = \Phi^{-1}[1-\alpha/2]$ and $\Phi[x] = P(Z \leq x)$ is the standard normal cumulative distribution function. We find that $|I| = |H_0|$ when the sample size confers 50% power to detect δ. With 80% and 90% power, we have $|I| = 0.7|H_0|$ and $|I| = 0.6|H_0|$, respectively, when $\alpha = 0.05$. It follows that the power has to drop below 16% for the correction factor to be triggered.

Remark 5. In future work, we intend to use 1/8 likelihood support intervals for the basis of our second-generation *p*-values. This is easily achieved with standard software by using a 96% CI from a normal approximation when the underlying sampling distribution is symmetric (which is most settings) [21].

Remark 6. It is instructive to see what happens as the indifference zone for fold change shrinks. Only 264 genes have Bonferroni corrected *p*-values less than 0.05, while 1233 genes have empirical false discovery rates (q-values) less than 0.05. There are 2028 genes with raw *p*-values less than 0.05. The ordered raw *p*-values are displayed in black along with Bonferroni adjusted *p*-values red and FDR/q-values (blue). Here $\alpha = 0.05$ in all cases. This is depicted in Supplementary Figure S1. The indifference zones are $\delta = 0.3, 0.1, 0.05, 1 \times 10^{-6}$. The second-generation *p*-values converge to $I(P > 0.05)/2$. The figure makes it clear second-generation *p*-values are doing something different than a routine multiple comparisons adjustment. Standard adjustments are made in *p*-value space where the effect size and variance are confounded. In contrast, second generation *p*-values are effectively making adjustments based on the observed effect size and screening out the results that are more likely to be false discoveries, i.e. the significant effects that are also null or practically null effects.

Remark 7. Figure S2 shows how the second-generation *p*-values were computed to color the rug plot. The estimated survival differences are plotted with their confidence interval and the indifference zone (shaded region). The confidence interval on the difference in survival rates could be computed using asymptotic methods or a simple bootstrap. Here we used the variance of the predictions from a cox proportional hazard model and assumed the two groups were independent. An alternative approach would be to estimate the baseline hazard using some other non-parametric method.

Remark 8. The 2x2 table examines a binary exposure's association, say smoking, with a binary outcome, say lung cancer. Imagine 100 smokers and 100 non-smokers, where 65 smokers and 50 non-smokers developed lung cancer. This is displayed in Table S1.

Table S1: Mock outcomes from a cohort study

	Outcome	
Exposure	**Lung CA**	**No Lung CA**
**Smoker**	65	35
**Non-Smoker**	50	50

The odds ratio of 1.86 measures the association between smoking and cancer. Here we have,

$$or = \frac{P(cancer|smoker)P(nocancer|nonsmoker)}{P(cancer|nonsmoker)P(nocancer|smoker)} = \frac{65 \times 50}{35 \times 50} = 1.86$$

Statistical computations are usually done on the natural logarithm scale, yielding a log odds ratio of 0.62 and a 95% CI of 0.05 to 1.19. The traditional *p*-value of 0.032 indicates statistical significance, rejecting the null hypothesis that the log odds ratio is 0. However, with an interval null of -0.1 to 0.1 (a 10% change in the odds ratio), we have $p_\delta = 0.044 = \frac{(0.1-0.05)}{(1.19-0.05)}(1)$ indicating the data are inconclusive and only suggestive of a real effect. The magnitude of an inconclusive second-generation *p*-value can vary slightly when the effect size scale is transformed. However definitive findings, i.e. a p_δ of 0 or 1, are *not* affected by the scale changes.

Like traditional *p*-values, the scale of the effect size can matter for reporting findings. Consider the contingency table example in section 3.1. Using the natural odds ratio scale (anti-log), the 95% CI is 1.05 to 3.29. The second-generation *p*-value is now slightly less at $p_\delta = 0.024 = \frac{(1.11-1.05)}{(3.29-1.05)}(1)$. While the conclusion is essentially the same, the degree to which the data are deemed "inconclusive" varied slightly. Importantly, a p_δ of 0 or 1 will *not* be affected by

monotonic scale changes (virtually all inferential transformations are monotonic). When a second-generation *p*-value indicates complete (in)compatibility with the null hypothesis, the results are invariant to the analysis scale.

Remark 9. Data on glycohemoglobin from the 2009-2010 National Health and Nutrition Examination Survey (NHANES), was designed to assess the health and nutritional status of adults and children in the United States [27,28]. Hemoglobin A1c (HbA1c), is a measure of the amount of glucose bound to hemoglobin in red blood cells and is a popular biomarker in diabetes and cardiovascular disease research.

Suppose we have the following linear regression model for HbA1c

$$\begin{aligned} HbA1c = \beta_0 + \beta_1 age + \beta_2 sex + \beta_3 race \\ + \beta_4 weight + \beta_5 waist + \beta_6 triceps + \epsilon \end{aligned} \tag{S3}$$

with independent errors $\epsilon \sim N(0, \sigma^2)$. Taken together, weight, waist size, and triceps thickness represent the impact of body size on HbA1c. We can assess the contribution of body size to this model and remove these predictors if they do not contribute sufficiently. This is usually posed as a problem of determining if the parameter vector $[\beta_4, \beta_5, \beta_6]$ is sufficiently close to $[0,0,0]$.

We could compute the second-generation *p*-value for the three-dimensional vector, but this requires specifying a three-dimensional interval null and obtaining simultaneous confidence intervals, i.e. a CI for the entire vector as opposed to three independent CIs for each element. A more elegant approach is examining how much the explained variance decreases when body size is removed from the model. That is, how different are the coefficients of determination (R^2)

between the full and reduced model, say $R_f^2 - R_r^2$. Routine alternatives are described below. A CI for this difference is easily bootstrapped. Algina et al. [29] advocate for the delta method approximation by Alf [30], while Smithson [31] uses the non-central F-distribution.

The three 95% CIs were 0.0231 to 0.0427 (BCa bootstrap), 0.0251 to 0.04107 (delta method), and 0.0246 to 0.0405 (non-central F). Suppose our null interval for describing an impactful contribution is $H_0: 0 \leq R_f^2 - R_r^2 \leq 0.025$. The resulting second-generation *p*-values are 0.097, 0, and 0.024. Notice that the choice of confidence interval method turns out to be important, so this should be carefully considered before examining the data. The two conservative methods indicate the data are only suggestive of a contribution and still inconclusive. If, however, we consider a more stringent criterion, such as $H_0: 0 \leq R_f^2 - R_r^2 \leq 0.05$, all three second-generation *p*-values would be 1, indicating the improvement in R^2 is less than 5%.

It is sometimes helpful to benchmark the reduction in explained variation against the total amount of unexplained variation that results when only the reduced model is fit. This has a direct tie to the tradition partial f-test or 'chunk-test'. This can be obtained by simply re-scaling the obtained CIs by $1 - R_r^2$ so that the estimand of interest is $R_f^2 - R_r^2/(1 - R_r^2)$, which is just the squared partial correlation $R_{f.r}^2$. The routine ANOVA F-statistic for this comparison is just $R_{f.r}^2(df_f)/(1 - R_{f.r}^2)(df_f - df_r)$. See Smithson [31] for details.

Remark 10. **Statistical and frequency properties of second-generation *p*-values**

There are three cases to consider: the probability data are compatible with the alternative, $P(p_\delta = 0)$, the probability data are compatible with the null, $P(p_\delta = 1)$, and the probability data are inconclusive $P(0 < p_\delta < 1)$. Note that we have three potential outcomes to consider instead of just two ("Reject the null" or "Fail to reject the null"). In Remarks 11 through 18, we examine the statistical properties of second-generation *p*-values when the sampling distribution of the estimator can be approximated by a normal distribution. This scenario covers a large majority of statistical applications, including methods of moments and maximum likelihood estimation, as well as common non-parametric estimators in large samples.

Remark 11. **Distributional assumptions:** Let $\hat{\theta}_n$ be an estimator of parameter θ. We consider the case where the sampling distribution is $\sqrt{n}(\hat{\theta}_n - \theta) \overset{A}{\sim} N(0, V)$ where the variance V is known or can be readily estimated. This scenario reflects the core behavior of a large majority of statistical applications, such as methods of moments, maximum likelihood estimation, and some common non-parametric estimators in large samples, i.e., U-statistics.

Remark 12. **Observing data compatible with the alternative hypothesis:** How often will a given set of data indicate compatibility with the alternative hypothesis? This probability, $P(p_\delta = 0)$, is analogous to power. Since p_δ is 0 only when the intersection between the intervals is the empty set, it follows that

$$P_\theta(p_\delta = 0) = \Phi\left[\frac{\sqrt{n}(\theta_0 - \delta)}{\sqrt{V}} - \frac{\sqrt{n}\theta}{\sqrt{V}} - Z_{\alpha/2}\right] + \Phi\left[-\frac{\sqrt{n}(\theta_0 + \delta)}{\sqrt{V}} + \frac{\sqrt{n}\theta}{\sqrt{V}} - Z_{\alpha/2}\right] \qquad \text{(S4)}$$

where θ_0 is the point null hypothesis and θ is the 'true' data generating hypothesis. As expected, the 'power curve' is a function of δ, the indifference zone margin.

When graphed, it looks like a power curve that was cut in half and pulled apart. Figure S3 displays the power for an interval null hypothesis of the form $H_0: \theta_0 - \delta \leq \theta \leq \theta_0 + \delta$, which was graphed for $\delta = 0, 0.03, 0.5, 1$. The zero origin on the x-axis represents θ_0. The loss in power is understandable. It is no longer sufficient to just rule out θ_0, we must rule out all of the null hypotheses. This effectively changes the anticipated effect size from $\theta_{alt} - \theta_0$ to $\theta_{alt} - \theta_0 - \delta$ which accounts for the power loss. Alternatives in the indifference zone are null hypotheses and the curve over that section of the x-axis represents the usual Type I Error rate. It should be clear from the graph that the maximum Type I error rate for second-generation *p*-values is bounded by α at all sample sizes (Remark 13 in S1). Type I Error rates near α occur when the true hypothesis is near or on the edge of the indifference zone.

Remark 13. When $\theta = \theta_0$, Equation S4 is analogous to the Type 1 Error rate which reduces to

$$P_{\theta_0}(p_\delta = 0) = 2\Phi\left[-\frac{\sqrt{n}\delta}{\sqrt{V}} - Z_{\alpha/2}\right] \qquad \text{(S5)}$$

Note the dependence on the sample size n and δ. Hence, the Type I Error rate is bounded above by $2\Phi\left[-Z_{\alpha/2}\right] = \alpha$. Moreover, it shrinks to 0 as the sample size approaches infinity, for any given $\delta > 0$. When $\delta = 0$, we recover the usual Type I Error rate.

Remark 14. **Comparison with a Bonferroni adjustment:** Figure S4 displays $P(p_\delta = 0)$ using an indifference zone of -0.3 to 0.3 similar to that from the Leukemia microarray example. For

comparison, we added the power curves from a single classical hypothesis test and from a Bonferroni correction procedure with 10, 1000, and 7128 comparisons. With 10 comparisons and a fixed variance, the Bonferroni and second-generation *p*-values have virtually identical operating characteristics (See Remark 16 in S1 for discussion). As seen in the microarray example, the second-generation *p*-value outperforms the Bonferroni adjustment with a large number of heterogeneous comparisons. Second-generation *p*-values achieve the benefit of traditional multiple comparisons adjustments through the use of a scientific adjustment instead of an ad-hoc statistical adjustment.

Remark 15. Just because two statistical procedures have nearly identical frequency properties does not imply that they will yield the same findings. A case in point is the second-generation *p*-value and Bonferroni procedure with k=10 comparisons in our example. Their power curves are nearly the same (Figure 6), implying they will have similar Type I and II error rates and similar family wise error rates. However, as the Leukemia example demonstrates, these procedures result in very different findings. Hence the Bonferroni procedure with k=10 is no substitute for a second-generation *p*-value.

Remark 16. **Observing data compatible with the null:** An important advance of second-generation *p*-values is that they can indicate when the data are compatible with the null hypothesis. How often does this happen? The data are compatible with the null hypothesis when the interval null contains the entire interval estimate. When the width of the interval estimate is less than the width of the interval null hypothesis, i.e., $|I| < |H_0|$ or $\delta > Z_{\alpha/2}\sqrt{V/n}$, we have

$$P_\theta(p_\delta = 1) = \Phi\left[\frac{\sqrt{n}(\theta_0+\delta)}{\sqrt{V}} - \frac{\sqrt{n}\theta}{\sqrt{V}} - Z_{\alpha/2}\right] - \Phi\left[\frac{\sqrt{n}(\theta_0-\delta)}{\sqrt{V}} - \frac{\sqrt{n}\theta}{\sqrt{V}} + Z_{\alpha/2}\right] \qquad \text{(S6)}$$

Otherwise, $P_\theta(p_\delta = 1) = 0$. Also, when the point null is the 'true' hypothesis, i.e., $\theta = \theta_0$, (S6) reduces to

$$P_{\theta_0}(p_\delta = 1) = \begin{cases} \Phi\left[\frac{\sqrt{n}\delta}{\sqrt{V}} - Z_{\alpha/2}\right] - \Phi\left[-\frac{\sqrt{n}\delta}{\sqrt{V}} + Z_{\alpha/2}\right] & \text{for } \delta > Z_{\alpha/2}\sqrt{V/n} \\ 0 & \text{o.w.} \end{cases} \qquad \text{(S7)}$$

Notice that when $\delta = Z_{\alpha/2}\sqrt{V/n}$, this expression is 0 because $P_{\theta_0}(\hat{\theta}_n = \theta_0) = 0$ by definition. Figure S5 displays $P(p_\delta = 1)$ as a function of the indifference zone margin δ when the sample size is small (right panel) and when the sample size is large (left panel). For very small indifference zones, it is virtually impossible to observe enough data to demonstrate compatibility with the null hypothesis. For large indifference zones, the data indicate compatibility with the null hypothesis most often when the true hypothesis is near the middle of the interval null.

Remark 17. **Observing data that are inconclusive:** Perhaps the scourge of any study is inconclusive results. Here we detail the probability that the second-generation *p*-value is inconclusive. The probability of observing data that are inconclusive is:

$$\begin{aligned} P_\theta(0 < p_\delta < 1) = 1 &- \Phi\left[\frac{\sqrt{n}(\theta_0-\delta)}{\sqrt{V}} - \frac{\sqrt{n}\theta}{\sqrt{V}} - Z_{\alpha/2}\right] - \Phi\left[-\frac{\sqrt{n}(\theta_0+\delta)}{\sqrt{V}} + \frac{\sqrt{n}\theta}{\sqrt{V}} - Z_{\alpha/2}\right] \\ &- \Phi\left[\frac{\sqrt{n}(\theta_0+\delta)}{\sqrt{V}} - \frac{\sqrt{n}\theta}{\sqrt{V}} - Z_{\alpha/2}\right] + \Phi\left[\frac{\sqrt{n}(\theta_0-\delta)}{\sqrt{V}} - \frac{\sqrt{n}\theta}{\sqrt{V}} + Z_{\alpha/2}\right] \end{aligned} \qquad \text{(S8)}$$

when $\delta > Z_{\alpha/2}\sqrt{V}/\sqrt{n}$ and

$$P_\theta(0 < p_\delta < 1) = 1 - \Phi\left[\frac{\sqrt{n}(\theta_0 - \delta)}{\sqrt{V}} - \frac{\sqrt{n}\theta}{\sqrt{V}} - Z_{\alpha/2}\right] - \Phi\left[-\frac{\sqrt{n}(\theta_0 + \delta)}{\sqrt{V}} + \frac{\sqrt{n}\theta}{\sqrt{V}} - Z_{\alpha/2}\right] \quad \text{(S9)}$$

otherwise. Figure S6 displays this behavior. When the indifference zone is small relative to the intended precision, and the true hypothesis is in or near the indifference zone, the probability of inconclusive results is high. As the indifference zone widens, the probability of inconclusive results remains high at its edges when the truth is also at the edges. But the probability drops rapidly near the middle of the zone when such results would indicate compatibility with the null hypothesis. The take home message here is that data will tend to be inconclusive when the truth is near the edges of the indifference zone. The practical solution to this problem is to use an indifference zone that is neither too large nor too small. But, of course, this is much easier said than done.

Remark 18. The delta-gaps are: Here $\delta = \log_{10} 2 = 0.3$ and the null interval is $[-0.3, 0.3]$. Gene #2288 has a 95% CI of 2.11 to 2.87 on the $\log_{10}$ scale with a delta gap of $6.03 = (2.11 - 0.3)/0.3$. Gene #3252 has a 95% CI of 1.22 to 1.64 with a delta gap of $3.07 = (1.22 - 0.3)/0.3$.

Remark 19. Both the false discovery rate (FDR), $P(p_\delta = 0|H_0)$, and the false confirmation rate (FCR), $P(p_\delta = 1|H_1)$, in (4) converge to zero as the sample grows because the probabilities, $P(p_\delta = 0|H_1)$ and $P(p_\delta = 1|H_0)$, converge to zero. This does not happen for the FDR in hypothesis testing, which converges to $\alpha/(\alpha + r)$, because the Type I Error rate is held constant at α. So design choices in this regard are consequential.

Remark 20. In the example used in the paper the FDR and FCR for second-generation *p*-values are smaller than their hypothesis testing counterparts. This is generally true for the FDR when the multiple comparisons being made have varying standard errors. However, it is possible for the FCR to be larger than the false non-discovery rate. This happens for hypotheses inside the null interval when the sample size is very large. As such is it not of consequence. By design, hypotheses within the indifference zone are not detectable by second-generation *p*-values. We believe this can be addressed by allowing the null interval to shrink at a rate slower than the interval estimate, but this will be detailed elsewhere. Figure S7 displays the FDR and FCR as the sample size changes. Note that the FCR is undefined in the first plot because the sample size is too small to permit nesting of the interval estimate in the interval null hypothesis.

Remark 21. In the pathological case when the variance across the multiple comparisons is constant, it is possible to select a δ such that the second-generation *p*-value's FDR would match the FDR of a Bonferroni correction. This connection highlights how the second-generation *p*-value outperforms the Bonferroni in a general setting. To have the same FDR as the Bonferroni, it would need to use a δ that depends on the standard error of each comparison. This is precisely what the second-generation *p*-value avoids, allowing it to detect large effects with relatively larger standard errors and while avoiding clinically meaningless effects that have relatively small standard errors.

References and Notes:

27. https://www.cdc.gov/nchs/nhanes/ or http://biostat.mc.vanderbilt.edu/wiki/Main/DataSets
28. https://pophealthmetrics.biomedcentral.com/articles/10.1186/s12963-015-0041-5
29. Algina J, Keselman HJ, Penfield RD. (2010). Confidence Intervals for the Squared Multiple Semipartial Correlation Coefficient. *Educational and Psychological Measurement*, 70(6): 926-940.
30. Alf EF and Graf RG. (1999). Asymptotic confidence limits for the difference between two squared multiple correlations: A simplified approach. *Psychological Methods*, 4: 70-75.
31. Smithson M. (2001). Correct confidence intervals for various regression effect sizes and parameters: The importance of non-central distributions in computing intervals. *Educational and Psychological Measurement*, 61(4): 605-632.

Figures:

Figure S1: *First and second-generation p-values plotted versus their ranking (number of rejected hypotheses). The black line gives unadjusted p-values, the blue line gives p-values after FDR adjustment, and the red line gives p-values after Bonferroni adjustment. Here* $\alpha = 0.05$ *in all cases. The green points are the second-generation p-values that result from the indifference zone in the title. Note the indifference zone narrows with* $\delta = 0.3, 0.1, 0.05, and\ 1 \times 10^{-6}$.

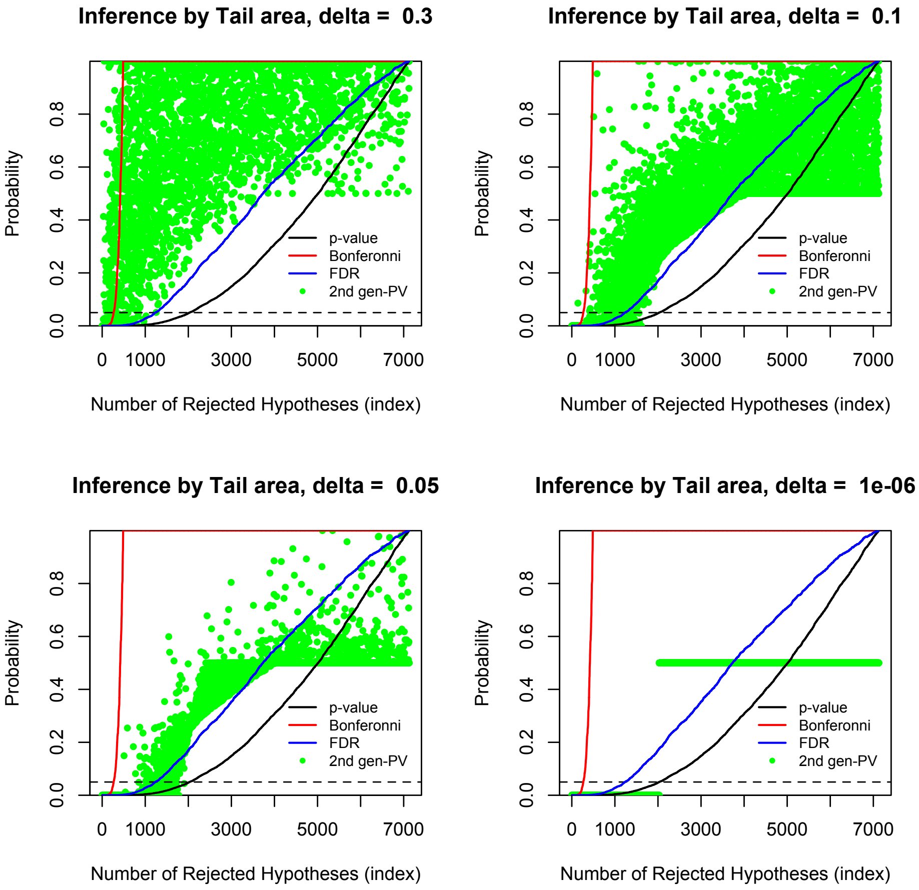

Figure S2: *The difference in survival fraction (black line) for in patients with advanced lung cancer from the North Central Cancer Treatment Group study. Red dashed lines are 95% confidence intervals. The indifference zone of +/- 5% is plotted in blue-grey. Rug plot on x-axis displays second generation p-values for the difference in survival time. Green ticks indicate incompatibility with null hypothesis; red indicate compatibility; gray indicate inconclusive results.*

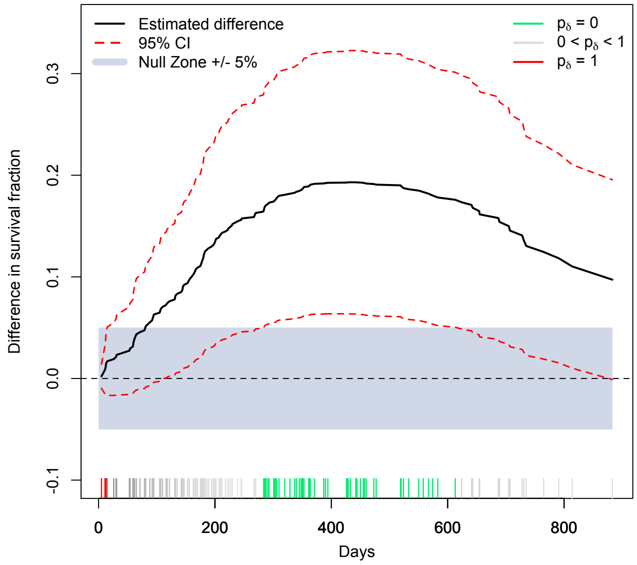

Figure S3: *The relationship between* $P(p_\delta = 0)$ *and various* δ*s. The black line represents the traditional case when* $\delta = 0$. *The orange line represents* $\delta = 1/30$, *the green line represents* $\delta = 1/2$, *and the blue line represents* $\delta = 1$.

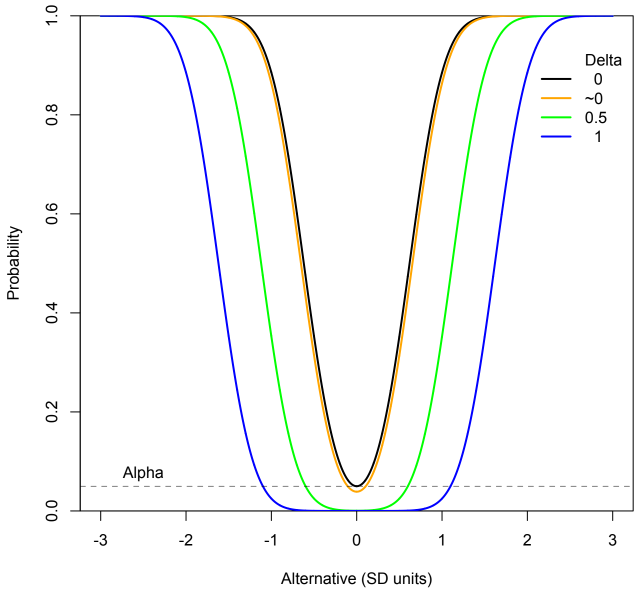

Figure S4: *Power curve comparisons of first-generation p-values (Black), second-generation p-values base on an indifference zone of* $\delta = 0.3$ *(similar to the Leukemia example), and Bonferroni adjusted p-values with k=10, 100, 7128 comparisons (red, orange, green).*

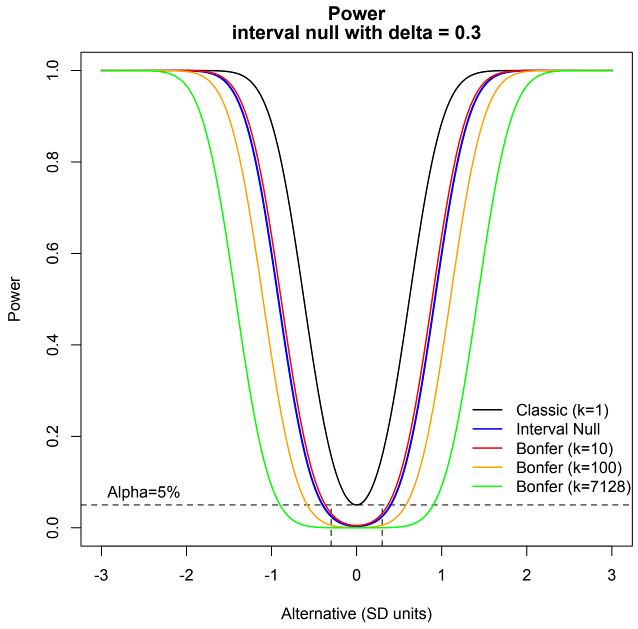

Figure S5: *The relationship between the probability of data supported compatibility with the null hypothesis,* $P(p_{\delta} = 1)$*, and various* δ*s. The black line represents* $\delta = 0$*, the traditional point null hypothesis. The orange line represents* $\delta = 1/30 \sim 0$*, a very small indifference zone relative to the observed precision. The green line represents* $\delta = 1/2$*, and the blue line represents* $\delta = 1$*, which are two larger indifference zone. The graph on the left has a smaller sample size while the graph on the right has a larger sample size.*

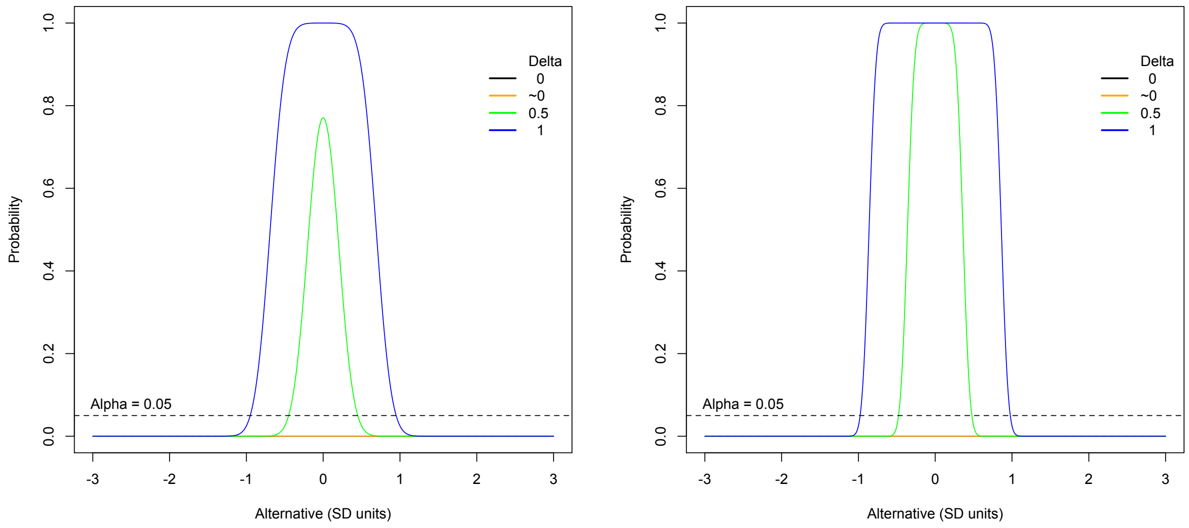

Figure S6: *The relationship between the probability of inconclusive results,* $P(0 < p_\delta < 1)$*, and various* δ*s. The black line represents* $\delta = 0.008$ *which is very close to the traditional point null hypothesis. The orange line represents* $\delta = 1/30 = 0.03$*, a very small indifference zone relative to the observed precision. The green line represents* $\delta = 1/2$*, and the blue line represents* $\delta = 1$*, which are two larger indifference zone. The graph on the left has a smaller sample size while the graph on the right has a larger sample size.*

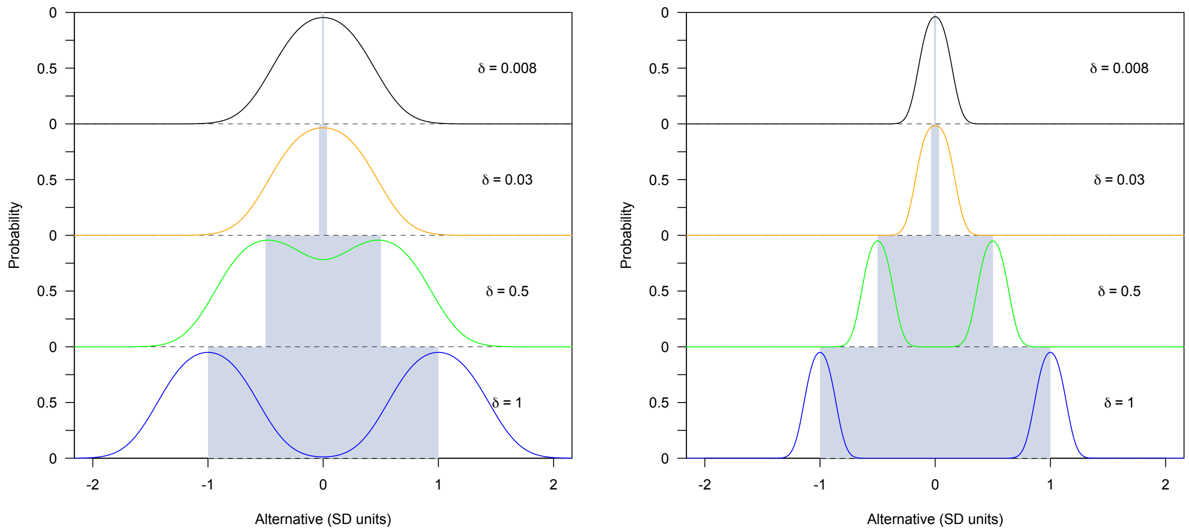

Figure S7: *Illustrations of the false discovery rate (red) and false confirmation rate (blue) for second-generation p-values (solid lines). The false discovery rate (red) and false non-discovery rate (blue) from a comparable hypothesis test are shown as dotted lines. This example uses* $r = 1, \alpha = 0.05, \delta = \sigma/2,$ and $n = 5, 20, 60, 100.$

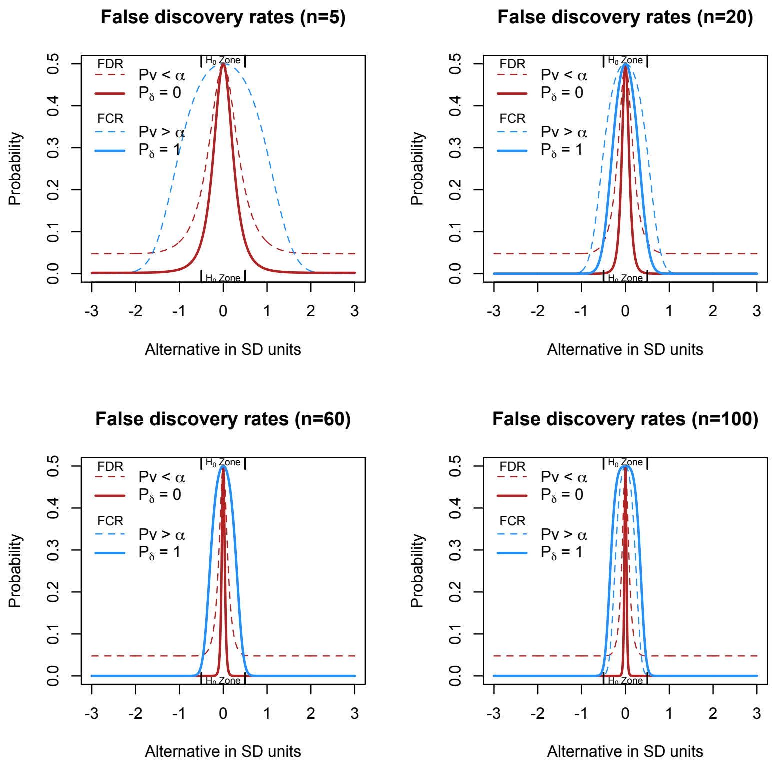

Supplement: S1 File — (PDF) [file pone.0188299.s001.pdf]
