# Supplementary material for: Second-generation p-values: Improved rigor, reproducibility, & transparency in statistical analyses
Source: PLoS One. 2018 Mar 22;13(3):e0188299. doi: 10.1371/journal.pone.0188299 (PMC5863943; doi:10.1371/journal.pone.0188299)
Supplement: S2 File — (PDF) [file pone.0188299.s002.pdf]

Supplementary Materials Part 2 (S2): Frequently Asked Questions

Title: Second-generation *p*-values: improved rigor, reproducibility & transparency in statistical analyses

To appear in PLOS ONE

Authors: Jeffrey D. Blume*, Lucy D'Agostino McGowan, William D. Dupont, Robert A. Greevy Jr.
*Correspondence to: j.blume@vanderbilt.edu

Contents:

Listing of Questions: Pages: FAQ 1
Answers: Pages: FAQ 2 – FAQ 6

Frequently Asked Questions (FAQ) about second-generation ***p-value*s**

Q.1: Why is it called a 'second-generation' *p*-value?
Q.2: How does one interpret a second-generation *p*-value?
Q.3: Is the 'second-generation' *p*-value a proportion or probability?
Q.4: Is the second-generation *p-value* the posterior probability of the null hypothesis assuming a non-informative prior?
Q.5: What is the second-generation *p-value* estimating?

Q.6: Why can't I interpret a traditional *p*-value in the way that a second-generation *p*-value is interpreted?
Q.7: Why is the second-generation *p-value* more useful than the simple report of the 95% confidence interval?
Q.8: Is it a problem that second-generation *p-values* of zero can be associated with different levels of precision?
Q.9: Do I need to use the same interval null if I want to compare second-generation *p-values*?
Q.10: Why do we need three regions for interpreting data?

Q.11: Why do we need to consider an interval null hypothesis?
Q.12: Why are very small differences, say near zero changes, between populations not scientifically meaningful?
Q.13: Who determines the width of the null interval?
Q.14: Why is the null interval uniform around the point null and not some other shape?
Q.15: How do we guarantee that the null interval would be defined before data were collected?

Q.16: Why have we been using point null hypotheses for so long?
Q.17: Can a classical p-value be computed for an interval null hypothesis?
Q.18: Why do I have to set the interval null before looking at the data?
Q.19: Can I report the smallest null interval for which the second-generation *p-value* is still zero?
Q.20: What is wrong with assessing scientific meaningfulness after the analysis is complete?

Q.21: Won't second-generation *p-values* be harder for non-statisticians to understand?
Q.22: Isn't the problem with traditional *p*-values that we have to choose an arbitrary cutoff? Can't this be fixed by finding the right cut-off?
Q.23: Is the precision of a single data point really relevant in determining the interval null width?
Q.24: We don't know much about second-generation *p-values*, so should we use it?

Q.1: Why is it called a 'second-generation' *p*-value?

Ans: Our proposed new metric is a conceptual generalization of the *p*-value that is rooted in the duality of confidence intervals and hypothesis testing. Hence the "second-generation" tag seems appropriate. Instead of checking to see if a singular null hypothesis is the interval, we now check to see how many of the practical representations of the null hypothesis are in the interval. We report how many of the best supported hypotheses are null hypotheses (e.g., all of them, $p_\delta = 1$, or none of them, $p_\delta = 0$).

Q.2: How does one interpret a second-generation *p*-value?

Ans: Second-generation *p*-values have a very natural interpretation: the fraction or proportion of data-supported hypotheses that are null hypotheses. Second-generation *p*-values are descriptive statistics that are intended to summarize the degree to which the study generated support for the null hypotheses or alternative hypotheses. A $p_\delta = 1$ or $p_\delta = 0$ indicates that the study has reached a natural stopping point.

Q.3: Is the 'second-generation' *p*-value a proportion or probability?

Ans: Second-generation *p*-values are proportions. They are not estimates of some unknown population quantity; they are not an estimate of the probability that the null hypothesis is true (neither is a first-generation *p*-value, for that matter). The *p* in p_δ is intended to stand for proportion not probability. It can be helpful to think of second generation *p*-values as an indicator of when the study has reached its goal.

Q.4: Is the second-generation *p*-value the posterior probability of the null hypothesis assuming a non-informative prior?

Ans: No. The posterior probability is P(Null | Data), which is not a second-generation *p*-value if only because the conditioning set is different.

Q.5: What is the second-generation *p*-value estimating?

Ans: Nothing. It is a descriptive statistic. It describes the proportion of the data-supported hypotheses that are null hypotheses. In large samples, the second-generation *p*-value converges to zero or one, and is thus best thought of as a marker of when an experiment reaches its predetermined goal.

Q.6: Why can't I interpret a traditional *p*-value in the way that a second-generation *p*-value is interpreted?

Ans: Because the theory of significance testing is quite clear: traditional *p*-values have a uniform distribution under the null hypothesis. Hence, the magnitude of non-significant *p*-values is indicative of nothing more than randomness; any number larger than α is inconclusive. It

is impossible, by design, for a traditional *p*-value to represent evidence for the null hypothesis. This is, in part, why replicating and interpreting non-significant *p*-values is so problematic. Under the null hypothesis *p*-values are just random variables that bounce around between 0 and 1.

Q.7: Why is the second-generation *p*-value more useful than the simple report of the 95% confidence interval?

Ans: It is more useful for providing a quick assessment of whether the experiment met is pre-determined goals. It saves time and is easier to comprehend than assessing the overlap between a 95% CI and unstated hypotheses of scientific interest. Second-generation *p*-values are not a replacement for 95% CIs. Confidence intervals are important in that they provide information on the range of effects and level of precision supported by the data.

Q.8: Is it a problem that second-generation *p*-values of zero can be associated with different levels of precision?

Ans: No. The second-generation *p*-value is not meant to replace the confidence interval, but rather to augment it. It is only seeking to convey if any null hypotheses were among those best supported by the data. The data can be imprecise and still exclude all null hypotheses. When comparing two second-generation *p*-values of zero, we recommend comparing their delta-gaps (the distance from interval null to interval estimate in SD units).

Q.9: Do I need to use the same interval null if I want to compare second-generation *p*-values?

Ans: Yes, this is needed as well as the same type of interval estimate if you want to compare magnitudes of second-generation *p*-values. Note this is similar to traditional *p*-values, which are not comparable unless they are based on the same sample size. When the second-generation *p*-value is zero, we recommend using the delta gap – the distance from interval null to interval estimate in δ units – as a way to rank second generation *p*-values that are zero.

Q.10: Why do we need three regions for interpreting data?

Ans: The most important thing we can do is not misrepresent inconclusive results. Ideally, every experiment would conclude with data that are only compatible with either the null hypothesis $(p_\delta = 1)$ or alternative hypothesis $(p_\delta = 0)$. But with finite sample sizes, this is often not possible. This is a major issue for classical *p*-values that is resolved by second-generation *p*-values.

Q.11: Why do we need to consider an interval null hypothesis?

Ans: Point null hypotheses are neither detectable nor a practical reality. Measurement devices have limited precision and many small non-zero changes are not actionable, consequential, or reproducible. As a result, for any experiment, there is actually a range of near null effects that are indistinguishable from the point null hypothesis and inferentially inconsequential. These are all null hypotheses and there is no reason to discriminate between them.

Q.12: Why are very small differences, say near zero changes, between populations not scientifically meaningful?

Ans: This depends on context. If the difference can be measured on an individual unit, then it can be meaningful and lead to action or intervention. Often, we find differences between populations are within the precision of the instrument. In that case, it is not clear if the difference is "real" or due to measurement error. For example, if the average income between two states were found to differ by one-half of one cent, would this be meaningful or actionable? What about one-tenth of one cent? There is always some point at which the measurement scale effectively becomes discrete.

Q.13: Who determines the width of the null interval?

Ans: The researcher sets this benchmark when designing the study. This is often implicitly done when power projections are completed. In applications such as clinical trials, this often is justified and discussed. But the researcher sets his or her own benchmark.

Q.14: Why is the null interval uniform around the point null and not some other shape?

Ans: The interval null is neither uniform nor any other shape. No distributional assumptions about the interval null are needed to use a second-generation *p*-value. Simplicity is the motivating factor here.

Q.15: How do we guarantee that the null interval would be defined before data were collected?

Ans: We can't. It is always possible to cheat. It is just as easy to cheat with *p*-values; it is called *p*-hacking. Protections such as pre-specified analysis plans will work similarly for second-generation p-values as they do for first generation p-values.

Q.16: Why have we been using point null hypotheses for so long?

Ans: Allowing the statistical procedure to pretend it has infinite precision is a welcome mathematical convenience. But the price of convenience is high, e.g. confusion as to whether statistical significance imparts a consequential finding. And when the assessment

of clinical or scientific significance is forgotten, ignored, or determined after looking at the data, the results are deemed suspect. This last concern is real; despite our best efforts, the definition of a meaningful effect tends to change after the data are observed.

Q.17: Can a classical *p*-value be computed for an interval null hypothesis?

Ans: Classical *p*-values for interval null hypotheses are undefined. This is because there exists a set of *p*-values, one for every null hypothesis. No single number summary of the *p*-value set captures, in general, how compatible the data are with the interval null hypothesis. It might be that the maximum *p*-value best tells the story in one case, while in another the minimum *p*-value or a weighted average of *p*-values is best. The reason for this is that the data may support some parts of the null set and not others.

Q.18: Why do I have to set the interval null before looking at the data?

Ans: This is good experimental practice: defining what success means before the experiment is conducted. Otherwise, the data are used twice: once to set the interval null hypothesis and once to check if the data are compatible with the interval null. This dual use of the data prevents the analysis from being confirmatory, increases the chances that the results are a false positive, and reduces the chances that the results can be reproduced.

Q.19: Can I report the smallest null interval for which the second-generation *p*-value is still zero?

Ans: Sure, but such an analysis would be considered exploratory. Instead, we suggest reporting the delta-gap (the distance from interval null to interval estimate in SD units) when the second-generation *p*-value is zero.

Q.20: What is wrong with assessing scientific meaningfulness after the analysis is complete?

Ans: The problem with checking scientific relevance after establishing statistical significance is that it allows after-the-fact rationalization, which reduces the rigor and reproducibility of published results. Requiring that the smallest effect of interest be specified up front, as is done with second-generation *p*-values, improves scientific rigor and reproducibility. In many applications, such as clinical trials, the interval null is already implicitly specified upfront for power projections.

Q.21: Won't second-generation *p*-values be harder for non-statisticians to understand?

Ans: Not in our experience. Non-statisticians readily acknowledge that there is always a null region of effects, and they readily accept an interval of hypotheses that are supported by the data. These are routine elements of current practice. The intersection of these two intervals follows naturally and is easy to conceptualize. Some understanding of confidence

intervals and hypothesis testing is necessary for power projections or for understanding their frequency properties. But an advanced level of understanding is not required for interpreting observed data with second-generation *p*-values.

Q.22: Isn't the problem with traditional *p*-values that we have to choose an arbitrary cutoff? Can't this be fixed by finding the right cut-off?

Ans: No. The problem is the metric itself. Choosing a different cutoff would not resolve issues with interpretation, computation or reproducibility. This is because adjustments like Bonferroni search for findings in *p*-value space where scientific meaningfulness is obfuscated. In contrast, second-generation *p*-values search for findings only in the space of scientifically meaningful results. Moreover, second generation *p*-values classify results into three categories (supporting the null hypothesis, inconclusive, supporting the alternative hypotheses), while traditional *p*-values use only two (inconclusive and supporting the alternative hypotheses).

Q.23: Is the precision of a single data point really relevant in determining the interval null width?

Ans: That is just one method of determining the width of the interval null hypothesis. It can be set in many ways. However, it makes little sense to search for differences between populations that cannot be measured in an individual. For example, what would it mean if a drug were found to reduce systolic blood pressure by 1 mmHG? If we gave that drug to an individual, the reduction in blood pressure would not be measurable nor is it considered clinical meaningful. Moreover, it would not be clear if the difference is due to measurement error or a real effect.

Q.24: We don't know much about second-generation *p*-values, so should we use it?

Ans: Yes. While there is certainly more to explore, this should not reduce enthusiasm for potentially unifying advance. Besides, second-generation *p*-values are essentially a formalization of current practice that is more likely to be reproducible and transparent.
